# Differentially charged isoforms of apolipoprotein E from human blood are potential biomarkers of Alzheimer’s disease

**DOI:** 10.1186/alzrt273

**Published:** 2014-07-14

**Authors:** Oscar Alzate, Cristina Osorio, Robert M DeKroon, Ana Corcimaru, Harsha P Gunawardena

**Affiliations:** 1 Systems Proteomics Center, School of Medicine, University of North Carolina at Chapel Hill, Chapel Hill, NC 27599, USA; 2 School of Medicine, Universidad Pontificia Bolivariana, Medellin, Colombia; 3 Department of Biochemistry and Biophysics, University of North Carolina at Chapel Hill, Chapel Hill, NC 27599, USA; 4 Current address: 108 Reynolds Medical Building, College of Medicine, Texas A&M Health Science Center, College Station, TX 77843-1114, USA

## Abstract

**Introduction:**

Alzheimer’s disease (AD) is the major cause of dementia among the elderly. Finding blood-based biomarkers for disease diagnosis and prognosis is urgently needed.

**Methods:**

We studied protein distributions in brain tissues, cerebrospinal fluid (CSF), and blood of AD patients by using proteomics and a new proteomic method that we call “2D multiplexed Western blot” (2D mxWd). This method allows us to determine in multiple samples the electrophoretic patterns of protein isoforms with different isoelectric points.

**Results:**

Apolipoprotein E (ApoE) displays a unique distribution of electrophoretic isoforms in the presence of AD and also a unique pattern specific to the *APOE* genotype.

**Conclusions:**

The isoelectric distribution of differentially charged ApoE isoforms was used to determine the presence of AD in a small group of samples. Further studies are needed to validate their use as predictors of disease onset and progression, and as biomarkers for determining the efficacy of therapeutic treatments.

## Introduction

More than 5.4 million people have Alzheimer’s disease (AD) in the United States, and it has been calculated that, by the year 2050, 11 to 16 million Americans will have the disease [1]. Characterizing the AD-specific proteome and its dynamics will increase our understanding of the initiation and the progression of this disease, potentially allowing the development of specifically targeted treatments and strategies to prevent its onset. When AD is found in patients younger than 60 to 65 years of age, it is usually referred as “early-onset familial AD” (EOFAD). These cases represent only an estimated 5% of all AD cases. Most AD cases occur after 60 to 65 years of age (identified as “late-onset AD”, or LOAD). The gene *APOE*, encoding the lipid-carrier protein apolipoprotein E (ApoE), has been established as a risk factor of developing LOAD [2-7].

ApoE has three polymorphic variants (ApoE2, ApoE3, and ApoE4) and is involved in several metabolic functions, including lipid transport [8,9]. In the search for blood-based biomarkers of AD with the capacity to distinguish AD from non-AD patients and also to distinguish the different *APOE* genotypes, we first investigated which proteins vary in the hippocampus of AD patients as a function of *APOE* genotype and AD status (that is, diseased versus nondiseased).

Our findings led us to investigate differential protein expression in the cerebrospinal fluid (CSF) of the same AD patients. Our results demonstrated charge-based changes in specific ApoE isoforms rather than changes in the expression level of the ApoE protein. We therefore developed a method to determine the expression patterns of these differentially charged protein isoforms in the blood of AD patients.

We demonstrate here that, in principle, differentially charged isoforms of ApoE from human blood may be used to distinguish AD patients from non-AD control subjects. This is a very important finding, because most of the AD biomarkers currently in use (such as the hyperphosphorylated tau protein, and the amount of Aβ_1–42_ peptide) are analyzed in the CSF [10-17], but blood is substantially easier to process, and its acquisition is less invasive.

Some of the biomarkers currently in use involve an extensive list of molecules such as cortisol, pancreatic polypeptide, insulin-like growth factor-binding protein 2, microglobulin, vascular cell adhesion molecule 1, carcinoembryonic antigen, matrix metalloproteinase 2, CD40, macrophage inflammatory protein 1α, superoxide dismutase, homocysteine, ApoE, epidermal growth factor receptor, calcium, zinc, and interleukin 17 [18]. When using gender, *APOE* genotype, and age as biomarkers, provided a specificity of 77% in the diagnosis of AD, and including some of the previous biomarkers, the specificity improved to 84% [18]. We predicted the correct AD status in 16 of 18 plasma samples (~89%) with just one molecule, with the advantage that our observation can also be used to determine the *APOE* genotype of the patient providing the sample. It is important to indicate that when the results are analyzed according the *APOE* genotype, just the AD state of all of the *APOE 3/3* samples was diagnosed correctly, whereas only 50% of the *APOE 4/4* was diagnosed correctly. This observation suggests the need for a larger sample population to be used as a reference panel, and indicates that our approach satisfies the diagnosis of the *APOE 3/3* samples.

## Methods

### General overview of the methodology

**Figure 1:**
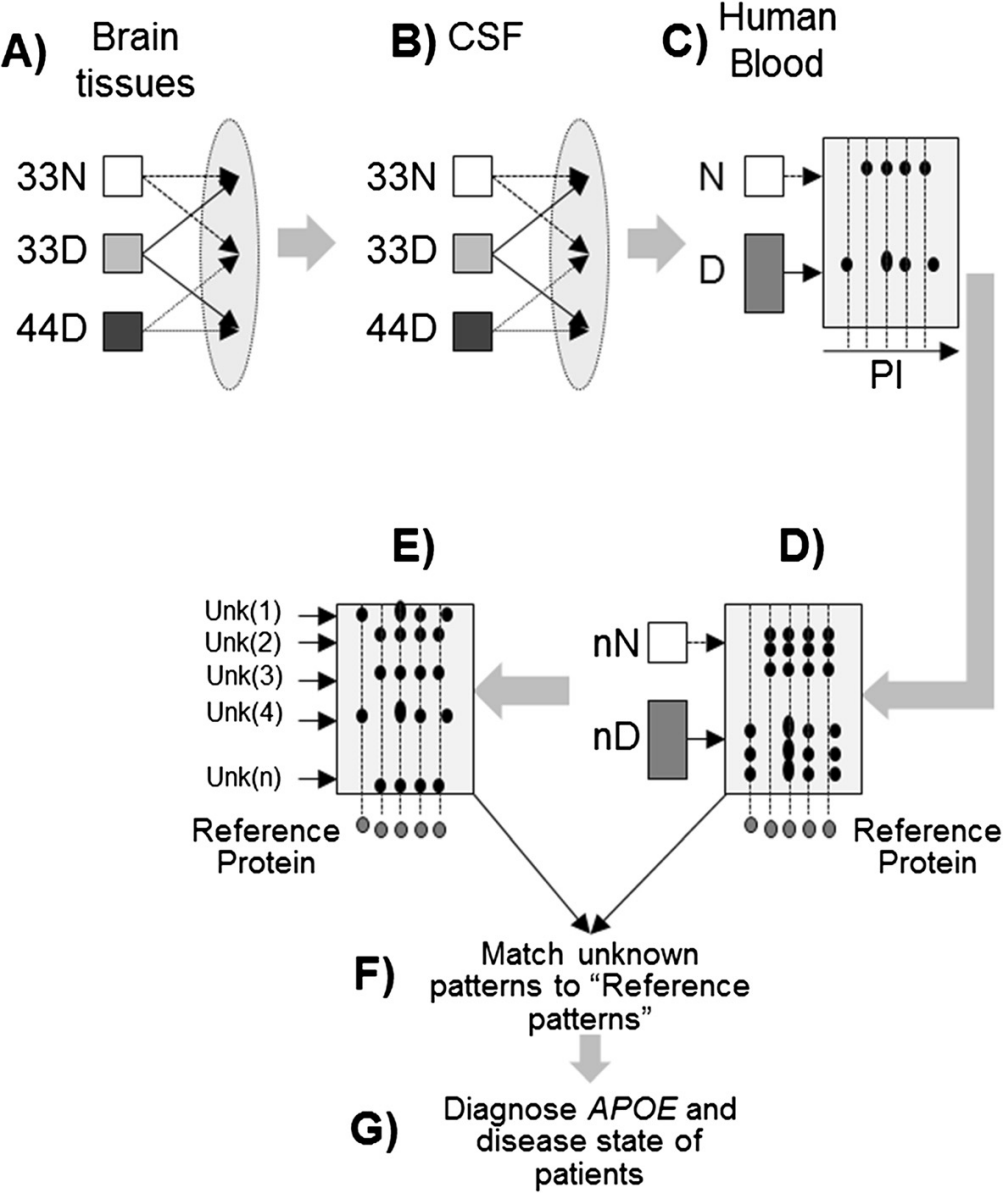
**Experimental design. (A)** Brain tissues from AD patients and non-AD control subjects were analyzed to determine differential protein expression. Three groups (non-AD, *APOE* 3/3 (33 N), AD *APOE* 3/3 (33D), and AD *APOE* 4/4 (44D)) were compared to each other. **(B)** A similar analysis was performed in the CSF samples of the same patients; **(C)** human blood serum from AD patients (D, *APOE* genotype-independent) and non-AD control subjects (N) were analyzed to investigate the distribution of protein isoforms of several proteins, including ApoA-1, ApoE, ApoH, and ApoJ. **(D)** The distribution of differentially charged protein isoforms of ApoE was analyzed in the blood of AD patients compared with non-AD control subjects by using the recombinant human ApoE protein as reference to determine charge-isoform distribution and migration patterns. **(E)** Similar experiments were carried out on a group of de-identified samples with unknown disease status. **(F)** The pattern of isoelectric distribution of the unknown subjects was compared with the reference panel developed in E. **(G)** The distribution of ApoE charge-isoforms was used as reference to predict the disease states and the *APOE* genotypes of the unknown samples.

Informed consent from the patients and/or their care providers for using the samples described in this study was obtained by the Bryan Alzheimer’s Disease Research Center (BADRC) of Duke University Medical Center, the Knight ADRC (KADRC) of Washington University School of Medicine in St. Louis (MO), and the Instituto Neurologico de Colombia (Colombian Neurological Institute, INDEC). Samples from AD patients are indicated with the letter D, while non-AD samples are indicated with the letter N. Samples from *APOE*3/3 or *APOE*4/4 diseased subjects are indicated 33D and 44D, respectively; non-AD *APOE*3/3 samples are indicated 33 N (Figure 1).

**Figure 2:**
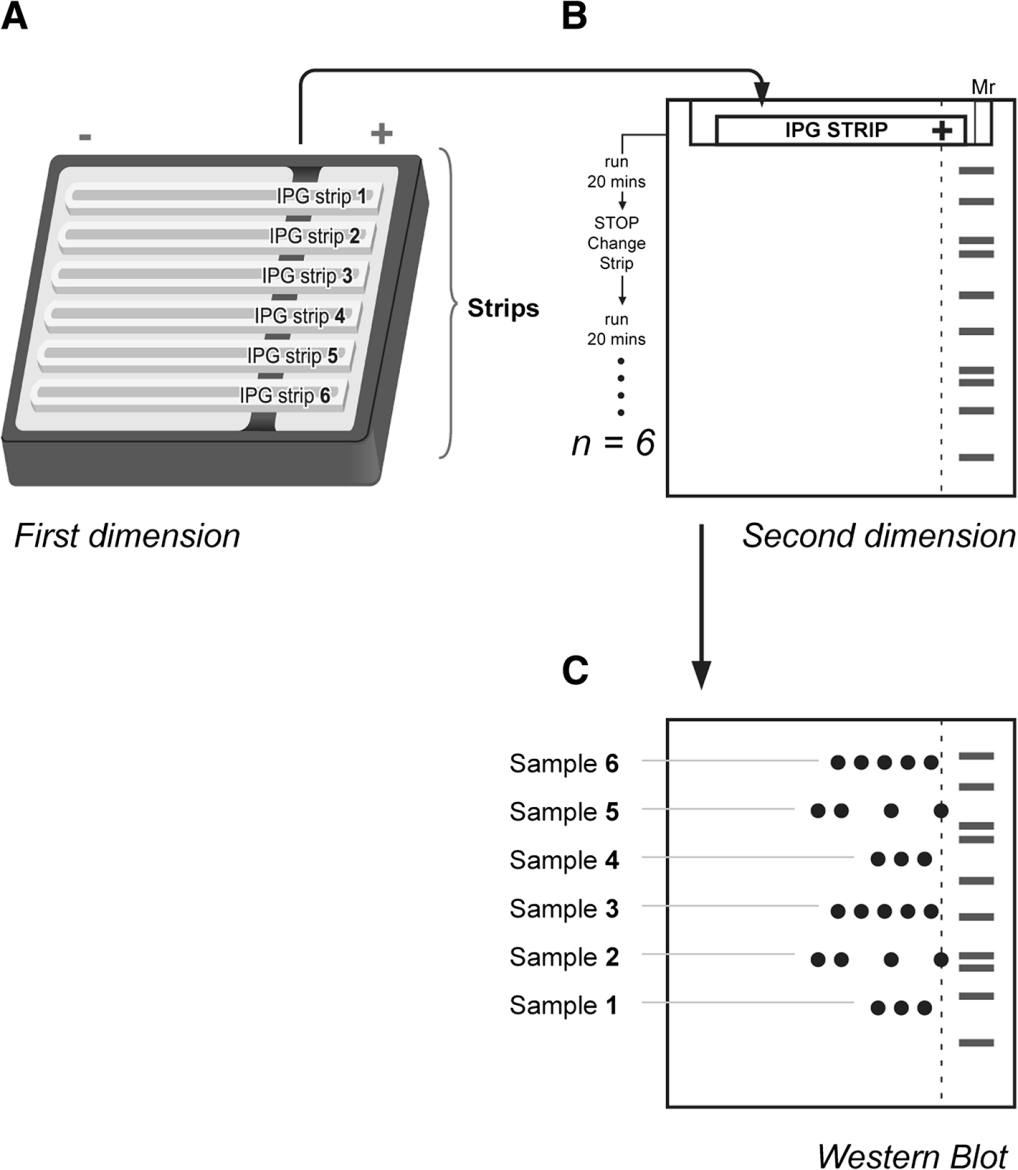
**Multiplexed 2D Western blot (2D mxWb) method. (A)** Proteins from several samples are separated simultaneously with isoelectric focusing. **(B)** The strips are then successively loaded onto a polyacrylamide gel. The first strip is run for about 10 to 20 minutes; after this time, this strip is removed, the gel surface is rinsed with d_ii_H_2_O, and the next strip is loaded and run for another 10 to 20 minutes; after this time, the procedure is repeated with the next strip until all of the strips are successively loaded in the SDS gel. **(C)** When all the strips have been run, the proteins are transferred onto a PVDF membrane and probed with specific antibodies. For the detection of ApoE, we used anti-goat ApoE antibody (CalBiochem) as the primary Ab and donkey anti-goat IgG-HRP as the secondary Ab.

The general method is summarized in Figure 1. (A) Hippocampi from AD patients and from non-AD control subjects were analyzed to determine differential protein-expression levels. From this analysis, we found sets of proteins unique to each disease state and unique to each *APOE* genotype. (B) The latter was also observed in the panels of proteins that displayed differential protein expression in the patient’s CSF compared with matched controls; (C) from the distribution of CSF proteins, it was found that for some proteins there were changes in the distributions of differentially charged protein isoforms. Therefore, the distribution of differentially-charged isoforms in the blood of AD patients was analyzed by using the method shown in Figure 2, which is explained in detail under section Two-dimensional multiplexed Western blotting (2D mxWb). (D) A general pattern for the differentially charged isoform distribution of ApoE was then sought in the blood of AD patients compared with age- and sex-matched controls. (E) the distribution of charge-isoforms of ApoE was determined in a “blind” group of unknown blood samples from several patients for whom no information was provided. (F) The distribution patterns of ApoE charge-isoforms from the unknown subjects were compared with the ApoE distribution patterns from the known AD patients; and (G) the matching of the charge-state distribution to the reference patterns was used to predict the disease state of the unknown samples.

### Sample procurement, protein extraction, and quantitation from CSF of deceased AD patients

Sample procurement, protein isolation, and proteomic analysis of human brain tissues have been previously reported [19]. CSF from deceased AD patients was acquired by the Kathleen Price Bryan Brain Bank of the BADRC, by following regulations of the Duke University Medical Center, as previously described [20], and was provided to us as de-identified samples (IRB exemption 6878-05-1R0ER, from Duke University Health System). These samples were centrifuged at 14,000 rpm to remove particulates, and the supernatant was filtered with a 20-μm filter. The CSF samples were prefractionated to enrich low-abundance proteins by removing high-abundance proteins by using the Multiple Affinity Removal System (MARS; Agilent Technologies, Wilmington, DE, USA), an antibody-based 100-mm affinity column, by following the manufacturer’s instructions. CSF (30 µL) was mixed with 120 μl of buffer A and injected into the column at a flow rate of 0.5 ml/min, by using the auto-sampler included with the 1100 Multidimensional Liquid Chromatography (MDLC) system (Agilent Technologies). The flow-through, containing the low-abundance proteins, was collected, and the high-abundance proteins that remained bound to the column were eluted with buffer B at a flow rate of 1 ml/min (buffers A and B are proprietary buffers included with the MARS depletion kit, and their composition is not disclosed by the manufacturer).

Proteins fractionated by affinity chromatography were reconcentrated with Agilent concentrator spin columns (5 kDa MWCO, 4 ml). Purified proteins were prepared as previously described [19,21]. Each de-identified sample, from patients whose ages were between 78 and 85 years, was classified only according to age, sex, *APOE* genotype (3/3 versus 4/4), and cognitive status (demented (D) versus nondemented (N)).

### Patient selection, sample procurement, and protein preparation from blood serum of AD patients

An additional group of samples used in the present study was obtained from a larger sample group previously used to study the correlation between type 2 diabetes mellitus and AD in the province of Antioquia, Colombia [22]. The samples were randomly selected from a group of 20 samples, all of which had been determined to be homozygous for *APOE3*[22]. The samples were collected by following norms and regulations approved by the ethics committees of the INDEC and the Universidad Pontificia Bolivariana (approved by the UPB research committee, 21/10/2010, as described in [22]). Because the methods used for this analysis involved working with human biologic specimens (that is, blood serum), an IRB exemption was filed and approved by the Office of Human Research Ethics of the University of North Carolina at Chapel Hill. The researchers involved in the present study received de-identified samples that were matched only by age and gender, and characterized as being from AD patients or from non-AD control subjects.

The selected samples were from six individuals (*n* = 6) who met the DSM-IVR (Diagnostic and Statistical Manual of Mental Disorders, 4th edition, American Psychiatric Association) criteria for AD diagnosis, and six control subjects who did not meet any DSM-IVR criteria for AD [22]. All AD patients were 65 years of age or older, with no relatives with an AD diagnosis or any type of dementia, and had no medical history of Parkinson disease, stroke, Huntington diseases, chronic subdural hematoma, and other conditions that could invalidate the diagnosis of AD [22]. The control group consisted of individuals older than 65 years of age of both genders, without a medical history of dementia syndrome or any other neurodegenerative disease. The individuals were matched by sex and age between the experimental and control groups.

Serum (40 μl) was aliquoted from each of the samples for protein purification. Sample clean-up used a chloroform-methanol extraction procedure [23], performed by mixing the serum sample with a 3:4:1 vol/vol mixture of d_ii_H_2_O, chilled methanol, and chloroform, and centrifuged for 15 minutes at 13,500 rpm. The samples were then suspended in lysis buffer [19,21], and the protein concentration of each sample was determined by using a BCA Protein Assay Kit (Thermo Fisher Scientific, Waltham, MA, USA). Protein quality was determined by 1D Western blot for all samples.

### Sample procurement and protein preparation from blood samples of patients with unknown AD diagnosis

Eighteen blood plasma samples, each containing 100 to 200 μl, from AD and control patients of the KADRC, were provided to us completely de-identified; the ethics committee of the KADRC, Washington University, approved all the methods involved in sample collection, preparation, and use for research. Because the samples were provided to us de-identified, an IRB exception was approved by the University of North Carolina. These samples were prepared as indicated earlier, and 10 μg of total protein from each sample was used for 2D multiplexed Western blotting (2D mxWb), as explained later.

### Cy-dye labeling and 2D gel electrophoresis for differential protein expression analysis

Differential protein expression in CSF from deceased AD patients and non-AD matched controls was determined by 2D Difference Gel Electrophoresis (2D DIGE), comparing all samples and determining protein-expression differences that correlated with *APOE* genotype and disease state [19,24]. Samples from 33D and 44D patients were used as the experimental groups and were compared with the 33 N control group. An internal control was created by pooling equal amounts of protein from all of the samples. This internal control was labeled with Cy2 and was applied to each gel, as previously described [19,24]. 2D DIGE was performed and analyzed, as previously described [21].

### Analysis of differential protein expression from CSF samples

The statistical significance of differences in protein expression was determined with GE Healthcare DeCyder 2D 7.0 software, as described [21], based on the Student t test by using *P* < 0.05 as threshold. Separate tests were performed comparing 33 N with 33D, 33 N with 44D, and 33D with 44D. The variability of the data and the Principal Component Analysis (PCA) were determined with DeCyder Extended Data Analysis (EDA) module [25]. The samples were compared as follows: (a) 33 N, containing six “non-AD” samples, all *APOE*3/3 genotype; (b) 33D, containing six “AD” patients, all genotype *APOE*3/3; and (c) 44D, containing six “AD” patients, all *APOE*4/4 genotype. PCA plots of each spot map were based on the expression profile of the selected proteins.

### Protein identification by mass spectrometry

Spots showing significant (*P* < 0.05) differential expression were excised from the gels, digested with trypsin [26], and identified by using MS and MS/MS on an Applied Biosystems AB 4800 MALDI TOF/TOF mass spectrometer at the Proteomics Center of the University of North Carolina at Chapel Hill, as previously described [27]. The proteins were identified by using the in-house GPS Explorer Mascot database [28].

### Two-dimensional multiplexed Western blotting

We developed this approach to determine variability in protein isoform distributions. Protein posttranslational modifications (PTMs) indicate addition, subtraction, and/or modifications of functional groups [29,30]. It is commonly found that such modifications alter the protein’s isoelectric point, the molecular weight, or both, making 2D gel electrophoresis an efficient method for analyzing PTMs [31]. Changes in the isoelectric point result in protein isoforms with different charges. The procedure for 2D mxWb is shown in Figure 2. Proteins from multiple samples were simultaneously separated by isoelectric focusing, as explained previously [19,31], by using one strip per sample (Figure 2A). For the experiments described here, we used an Ettan IPGphor II (GE Healthcare) with a capacity of up to 12 strips. After protein separation by isoelectric focusing, the strips from each sample are successively loaded onto a polyacrylamide gel to run the second dimension (Figure 2B). The first strip is run for approximately 20 minutes; after this time, this strip is removed, the gel surface is rinsed with d_ii_H_2_O, and a second strip is loaded and run for another 20 minutes. The total number of strips that can be run in a single 2D PAGE gel depends on several variables, including (a) the apparent molecular weight of the target protein, (b) the size and the density of the gel, and (c) the potential difference used to displace the proteins. To determine specific changes in a protein’s pI distribution, we used human recombinant ApoE protein (hr-ApoE; Fitzgerald Industries International, Acton, MS, USA; cat. 30R-AA016) as a reference. This protein is separated by IEF along with the other strips, and is loaded on the polyacrylamide gel by using the same procedures as for the target proteins.After running the second dimension, proteins are transferred to a PVDF membrane, and the proteins are probed with specific antibodies (Figure 2C). For detection of ApoE, ApoE-goat Ab (CalBiochem, La Jolla, CA, USA; cat. 178479) was used as the primary Ab, and donkey anti-goat IgG-HRP (Santa Cruz Biotechnology; cat. SC-2020) was used as the secondary Ab. From the Western blot analysis, it is possible to determine the migration patterns of the charge-isoforms for multiple samples in a single experiment.

### LC-MS/MS-based quantitative analysis of ApoE

Solubilized protein samples from human brain tissue were separated with 1D SDS (12%) PAGE electrophoresis with pre-cast gels (Bio-Rad, Hercules, CA, USA). The apparent protein molecular weight was determined with “Kaleidoscope” prestained protein standards (Bio-Rad). Gel strips for each sample were excised from the Coomassie-stained gel, and each gel strip was cut into 1 × 1-mm gel pieces and transferred into Axygen 1.7-ml tubes. Manual “in-gel” protein digestion was done as previously explained [26].

Extracted peptides were desalted by using PepClean C18 spin columns (Pierce, Rockford, IL, USA), used according to the manufacturer’s instructions, and resuspended in an aqueous solution of 0.1% formic acid. Identification of proteins was done by using reversed-phase LC-MS/MS on a 2D-nanoLC Ultra system (Eksigent Inc., Dublin, CA, USA) coupled to an LTQ-Orbitrap Velos mass spectrometer (Thermo Scientific, San Jose, CA, USA). The Eksigent system was configured to trap and elute peptides via an injection of ~250 f*M* sample. The trapping was performed on a 3 cm-long 100 μm i.d. C18 column, whereas elution was performed on a 15 cm-long 75 μm i.d., 5 μm, 300 Å particle ProteoPep II integraFrit C18 column (New Objective Inc, Woburn, MA, USA). Analytic separation of the tryptic peptides was achieved with a 70-minute linear gradient of 2% to 10% buffer B at a 200 nl/min. Buffer A is an aqueous solution of 0.1% formic acid, and buffer B is a solution of 0.1% formic acid in acetonitrile.

Mass spectrometric data acquisition was performed in a data-dependent manner on a hybrid LTQ-Orbitrap mass spectrometer. A full-scan mass analysis on an Orbitrap (externally calibrated to a mass accuracy of <1 ppm, and a resolution of 60,000 at m/z 400) was followed by intensity-dependent MS/MS of the 10 most abundant peptide ions. Collision-induced dissociation (CID)-MS/MS was used to dissociate peptides with a normalized collision energy of 35 eV, in the presence of He bath gas at a pressure of 1 mTorr. The MS/MS acquisition of each precursor m/z was repeated for 30 seconds and subsequently excluded for 60 seconds. Monoisotopic precursor ion selection (MIPS) and charge-state screening were enabled for triggering data-dependent MS/MS scans. Mass spectra were processed, and peptide identification was performed by using Mascot ver. 2.3 (Matrix Science Inc.) implemented on Proteome Discoverer ver 1.3 software (Thermo-Fisher Scientific). All searches were performed against a curated Human data base [32]. Peptides were identified by using a target-decoy approach with a false discovery rate (FDR) of 1% [33]. A precursor ion mass tolerance of 200 ppm and a product ion mass tolerance 0.5 Da were used, with a maximum of two missed tryptic cleavages [34]. Methionine oxidation was selected as a variable modification.

Spectral counting was performed on the Mascot DAT files by using ProteoIQ: ver 2.3.02 (NuSep Inc., Athens, GA, USA). Proteins identified with LC-MS/MS were subjected to “probability-based” confidence measurements by using an independent implementation of the statistical models Peptide and Protein Prophet deployed in Proteo IQ [35,36]. Protein hits were filtered with a probability of 0.5 and a Mascot identity with a significant score cut-off greater than 26. Identified proteins were analyzed for potential posttranslational modifications.

## Results

### Proteomic analysis of CSF shows that pI distributions are altered in AD patients as a function of the *APOE* genotype

**Figure 3:**
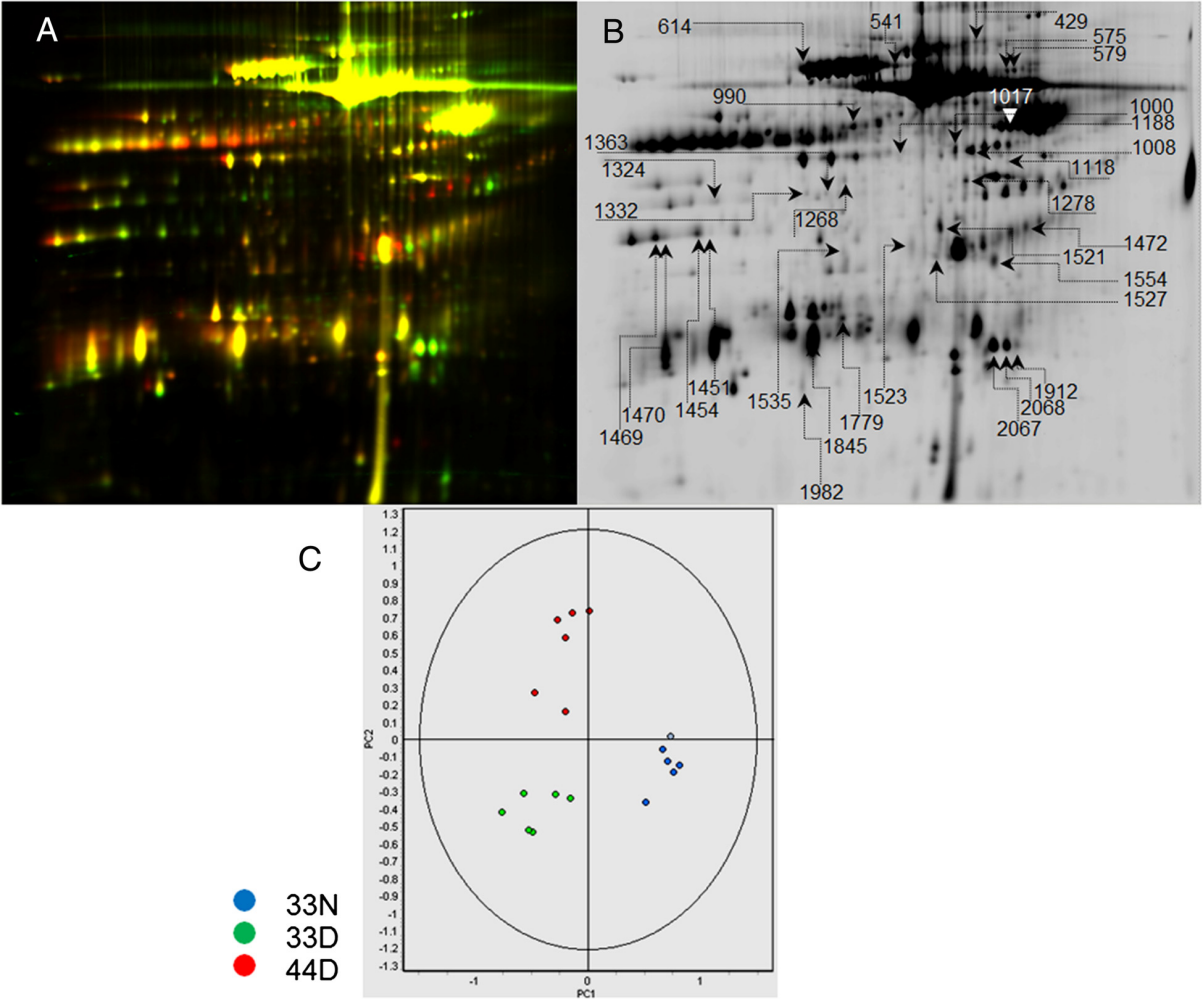
**Representative 2D DIGE gel of CSF proteins and PCA of brain proteins. (A)** Low-abundance proteins from CSF of AD patients and control subjects were compared, with control samples labeled with Cy3 (Green) and AD samples labeled with Cy5 (Red). **(B)** Protein spots displaying significant different expression levels, which were successfully identified with mass spectrometry, are shown with the spot numbers automatically assigned by the DeCyder 2D software. **(C)** PCA analysis of all the proteins detected from brain tissues of AD patients and corresponding controls indicates that these are distinct experimental groups.

One hundred thirty-one spots were differentially expressed in at least one comparison as follows: 15 in the 33N versus 33D comparison; 37 in 33N versus 44D; 21 in 33D versus 44D; and 58 in N versus D (see Additional file 1). Based on spot size and intensity, 40 of these spots contained a sufficient amount of protein to be identified with MS. In total, 32 of the 40 spots were successfully identified (Table 1, Figure 3B, and Additional file 1). Protein spots 541 and 614 are pI variants (that is, charge-isoforms) of transferrin variant protein (PIR: Q53H26); spots 575 and 579 are pI variants of α1-B glycoprotein (PIR: P04217); spots 1332 and 1363 correspond to pI variants of aspartate aminotransferase (PIR: P17174); spots 1451, 1454, 1469, and 1470 represent pI variants of glyceraldehyde-3-phosphate dehydrogenase (PIR: P04406); spot numbers 1472 and 1523 are charge-isoforms of apolipoprotein J (also known as clusterin (PIR: P10909)); spots 1521 and 1527 are pI variants of ApoE4 (PIR: P02649); and spots 1912, 2067, and 2068 are pI variants of apolipoprotein A-1 (PIR: P02647). Changes in the expression levels of all of these proteins are shown in Table 1. Please note that other charged isoforms of these proteins may be present, in addition to those indicated here, but these were not selected, as they did not show changes in protein-expression levels.

**Table 1:** Human CSF proteins displaying differential expression in AD patients

**Spot number**	**Protein name/Protein ID/Uniprot Accession Number/RefSeq**	**33 N versus 33D**	**33 N versus 44D**	**N versus D**	**Molecular weight**	**pI**
429	Serum albumin, Chain A; ALBU_HUMAN P02768/NP_000468.1	-1.29	-1.37	-1.33	69,366.68	5.92
				*P* = 0.00034		
541	Transferrin variant; Q53H26_HUMAN Q53H26 /IP|00022463	-1.53	-1.50	-1.51	77,079.85	6.68
				P = 0.0011		
575	alpha 1-B glycoprotein; A1BG_HUMAN P04217/NP_570602.2	-1.22	-1.18	-1.20	54,272.56	5.58
				*P* = 0.0013		
579	alpha 1-B glycoprotein; A1BG_HUMAN P04217/NP_570602.2	-1.31	-1.31	-1.31	54,272.56	5.56
				*P* = 0.0015		
614	Transferrin variant; Q53H26_HUMAN Q53H26/IP|00022463	+1.70	+1.64	+1.67	770,79.85	7.04
				*P* = 0.0024		
990	Apolipoprotein H (beta-2-glycoprotein I) APOH_HUMAN P02749/NP_000033.2	+1.67	+1.60	+1.63	38,298.16	8.34
				*P* = 0.0021		
1000	Tubulin beta-2A chain; TBB2A_HUMAN Q13885/NP_001060.1	+1.29	+1.73	+1.51	49,906.67	4.78
				*P* = 0.0058		
1008	Keratin, type II cytoskeletal 1; K2C1_HUMAN P04264/NP_006112.3	+2.94	+2.62	+2.77	66,038.73	8.15
				*P* = 0.002		
1017	alpha-1-antitrypsin (SerpinA1); A1AT_HUMAN P01009/NP_000286.3	+1.86	+2.26	+2.06 *P* = 0.0021	46,739.55	5.37
1118	Pigment epithelium-derived factor (SerpinF1); PEDF_HUMAN P36955/NP_002606.3	+1.54	+1.99	+1.77	46,342.30	5.97
				*P* = 7.4E-05		
1188	ALB protein (growth-inhibiting protein 20), Isoform 2; ALBU_HUMAN P02768/NP_000468.1	+1.26	+1.23	+1.24	47,360.49	5.97
				*P* = 0.0065		
1268	Glutamine synthetase; GLNA_HUMAN; P15104/NP_001028216.1	-1.38	-1.55	-1.46	42,064.46	6.43
				*P* = 0.0025		
1278	Creatine kinase B-type; KCRB_HUMAN; P12277/NP_001814.2	-1.46	-1.40	-1.43	42,644.28	5.34
				*P* = 0.0077		
1324	Fructose-bisphosphate aldolase A; ALDOA_HUMAN P04075/NP_000025.1	-1.49	-1.53	-1.51	39,420.02	8.30
				*P* = 0.0074		
1332	Aspartate aminotransferase, cytoplasmic; AATC_HUMAN; P17174/NP_002070.1	-1.28	-1.49	-1.38	46,247.51	6.53
				*P* = 0.0099		
1363	Aspartate aminotransferase, cytoplasmic; AATC_HUMAN; P17174/NP_002070.1	-1.27	-1.41	-1.33	46,247.51	6.50
				*P* = 0.0003		
1451	Glyceraldehyde-3-phosphate dehydrogenase G3P_HUMAN P04406/NP_002037.2	-2.05	-1.75	-1.87	36,042.22	8.57
				*P* = 0.0002		
1454	Glyceraldehyde-3-phosphate dehydrogenase G3P_HUMAN P04406/NP_002037.2	-1.45	-1.42	-1.44	36,042.22	8.59
				*P* = 0.013		
1469	Glyceraldehyde-3-phosphate dehydrogenase G3P_HUMAN P04406/NP_002037.2	-1.58	-1.52	-1.55	36,042.22	8.63
				*P* = 0.00094		
1470	Glyceraldehyde-3-phosphate dehydrogenase G3P_HUMAN P04406/NP_002037.2	-1.80	-1.72	-1.75	36,042.22	8.67
				*P* = 0.0005		
1472	Clusterin; Apolipoprotein J; Complement-associated protein SP-40; CLUS_HUMAN; P10909/NP_001164609.1	+2.01	+2.01	+2.01	50,062.56	5.89
				*P* = 5.10E-05		
1521	Apolipoprotein E4; APOE_HUMAN P02649/NP_000032.1	+2.14	+3.34	+2.74	36,154.08	5.65
				*P* = 0.00023		
1523	Clusterin; Apolipoprotein J; Complement associated protein SP-40; CLUS_HUMAN; P10909/NP_001164609.1	+1.78	+2.17	+1.97	50,062.56	5.89
				*P* = 3.50E-06		
1527	Apolipoprotein E4; APOE_HUMAN P02649/NP_000032.1	+2.02	+2.42	+2.22	36,154.08	5.65
				*P* = 1.30E-05		
1535	Complement component 4A; C4A; Q5JNX2_HUMAN; Q5JNX2/IP|00643525	+1.85	+1.94	+1.89	~3,5000	6.43
				*P* = 7.10E-05		
1554	Transthyretin; TTHY_HUMAN; P02766/NP_000362.1	+2.05	+2.58	+2.32	15,887.03	5.52
				*P* = 0.00078		
1779	Ig Kappa chain C region; IGKC; IGKC_HUMAN; P01834/IP|00909649	-1.98	-2.14	-2.06	11,608.86	5.58
				*P* = 0.0061		
1845	Prostaglandin-H2 D-isomerase; cerebrin-28; PTGDS_HUMAN; P41222/NP_000945.3	+1.45	+1.51	+1.48	21,028.82	7.66
				*P* = 0.0077		
1912	Apolipoprotein A-1; ApoA1; APOA1-HUMAN; P02647/NP_000030.1	-1.78	-1.84	-1.81	30,777.83	5.56
				*P* = 0.0037		
1982	Superoxide dismutase [Mn], mitochondrial; SODM_HUMAN; P04179/NP_000627.2	+1.66	+1.62	+1.64	24,722.09	8.35
				*P* = 0.0011		
2067	Apolipoprotein A-1; ApoA1; APOA1-HUMAN; P02647/NP_000030.1	-1.95	-2.09	-2.02	30,777.83	5.56
				*P* = 7.40E-06		
2068	Apolipoprotein A-1; ApoA1; APOA1-HUMAN; P02647/NP_000030.1	-2.25	-2.24	-2.24	30,777.83	5.56
				*P* = 0.00035		

The spot number is assigned by default by the DeCyder 2D 7.0 software, and is indicated on the gel image (Figure 3B). The protein name and the protein ID are obtained from the protein database maintained at the Protein Information Resource server, Georgetown University [37].

The Uniprot accession number and the *RefSeq* number are protein identifiers retrieved from the Uniprot database based on the protein identification achieved by using mass spectrometry data [38]. Changes in protein expression are expressed as the log of the ratios between the corresponding spots’ volumes, and are automatically calculated with DeCyder 2D.

The protein’s isoelectric point (pI) and molecular weight (Mw) are associated with the protein ID in PIR [37].

### Charge-based distribution of differentially charged ApoE isoforms distinguishes AD from non-AD samples

**Figure 4:**
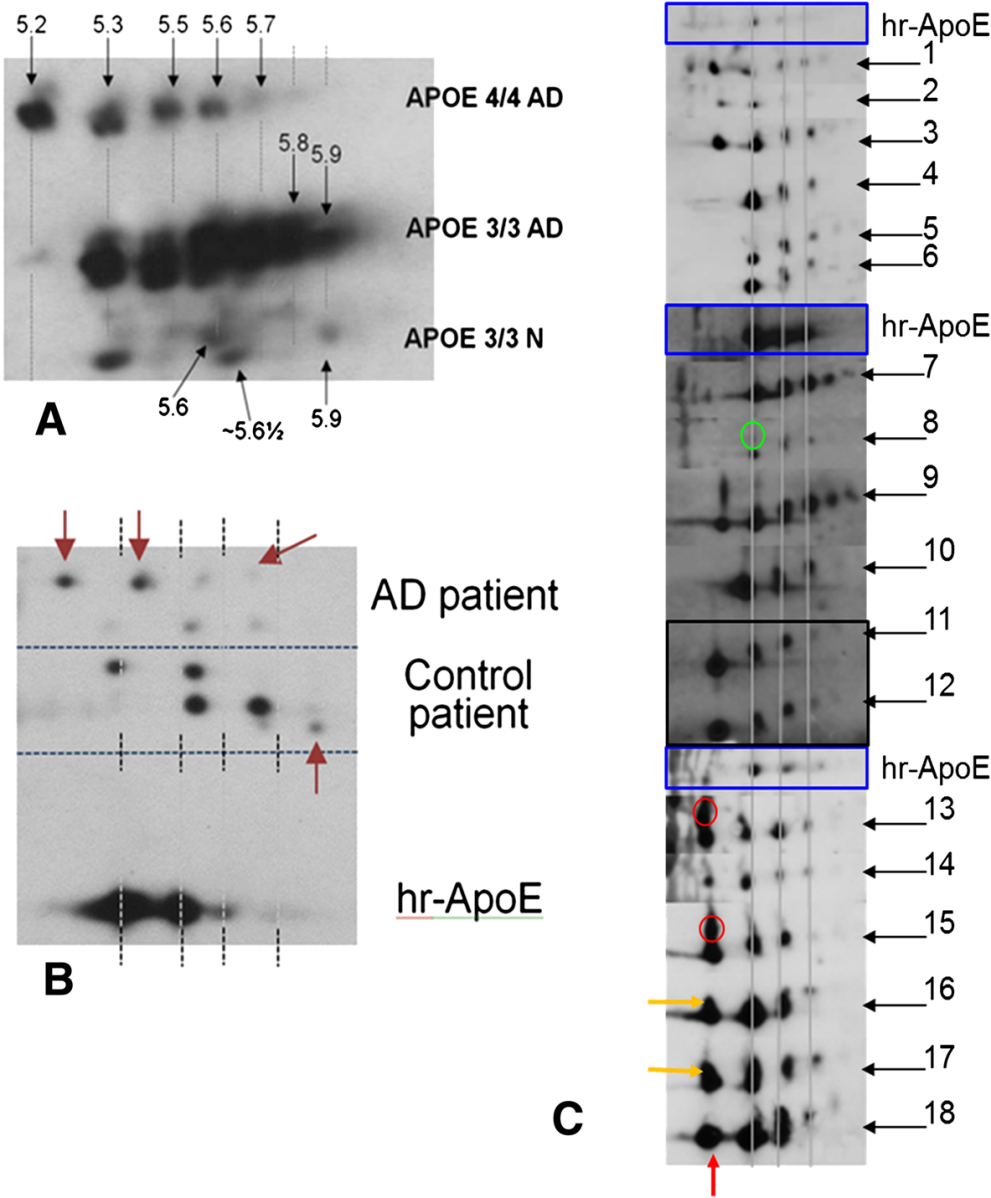
**Detection of ApoE charge-isoforms and prediction of AD. (A)** 2D mxWb analysis of the distribution of ApoE charge-isoforms in the hippocampus of AD patients and control subjects shows that the charge-isoform corresponding to a pI = 5.2 is unique to the 44D group; similarly, the spot at pI = 5.8 is more abundant in the E33 group; and the non-AD population displays fewer ApoE charge-isoforms and with lower expression levels. **(B)** Analysis of the ApoE charge-isoforms in the serum of AD patients shows that their migration patterns are unique to each population (indicated by the arrows). **(C)** The specific features of ApoE charge-isoform migration patterns are used to determine the disease state of each individual sample: the red arrow at the bottom indicates a feature (spot) that is uniquely found in patients having an ϵ4 gene. The yellow arrows on the left indicate features that are found in groups containing at least one ϵ3 along with one ϵ4, whereas the red circle shows a protein modification (“smear”) that has been found only in 44D samples. The green circle shows a feature that is unique to the 33D group. Each experiment was run in groups of six unknown samples and one reference protein, indicated as hr-ApoE.

Analysis of hippocampus from AD patients indicates that (a) the sets of proteins characterizing the diseased state and the *APOE* genotype of the AD patients represent distinctive experimental groups (PCA analysis, Figure 3C); (b) charge-isoforms of certain proteins can be differentially expressed without overall changes to the expression level of the whole protein [19]; (c) a pI variant of ApoE at pH 5.2 is found in the brains of *APOE*4/4 diseased patients and not found in *APOE* 3/3 AD or in the non-diseased controls (Figure 4A); and (d) the ApoE charge-isoform distribution in the non-AD controls is different from the distribution found in the sample from subjects with AD. This is a significant observation about the ApoE charge-isoform distribution in the human brain, but is, unfortunately, of low value for clinical biomarker research, considering that these are brain tissues. However, further analysis examining human blood from patients with diagnoses of AD (Figure 4B) shows that observations in hippocampal specimens are closely mirrored in the blood of the AD patients, which shows a unique distribution of differentially charged isoforms found only in AD patients, and not in the non-AD controls (Red arrows in Figure 4B point out spots that are unique to each sample). These observations were confirmed in six controls and six AD patients, resulting in a distinctive pI distribution that is summarized in Figure 4B.

### Plasma ApoE charge-isoform distribution may be able to predict AD

To determine the ability of our isoelectric isoform-distribution panel to predict AD, we performed 2D mxWb analysis of 18 de-identified samples (Figure 4C). The results of this analysis are shown in Table 2, which demonstrates that we were able to predict accurately the AD state in 16 of the 18 samples (88.9%), with only two of the samples, both of the *APOE*4/4 genotype, not predicting the diagnosis. The latter could be explained because we did not have any ApoE ϵ4/4 samples in our reference panel for comparison; the possibility also exists that our prediction was correct, but dementia was not yet apparent clinically.

**Table 2:** Prediction of disease state by using the panel of ApoE charge-isoforms

**Sample number**	***APOE *** **genotype**	**Disease state from clinic**	**Disease state predicted**
1	3/4	No dementia	No disease
2	3/4	No dementia	No disease
3	3/4	No dementia	No disease
4	3/3	No dementia	No disease
5	3/3	No dementia	No disease
6	3/3	No dementia	No disease
7	3/3	Dementia	Disease
8	3/3	Dementia	Disease
9	NR	Dementia	Disease
10	3/3	No dementia	No disease
11	4/4	No dementia	**Disease**^**a**^
12	4/4	No dementia	**Disease**^**a**^
13	4/4	Dementia	Disease
14	3/4	Dementia	Disease
15	4/4	Dementia	Disease
16	3/4	Dementia	Disease
17	3/4	Dementia	Disease
18	3/4	Dementia	Disease

Age range, 62.8 to 81.5 years; gender distribution, eight men, 10 women. The *APOE* genotype of the patients was unknown when the experiments were performed. NR, not reported.

^**a**^incorrectly predicted.

## Discussion

We analyzed the patterns of differential protein expression in the brains of AD patients, and we found that several proteins, including mortalin, display differential expression of individual isoforms, but not of the whole protein (that is, the single protein resulting from the sum of all the isoforms as detected by 1D Western blot) [19]. This observation was confirmed and extended when proteomic analysis of the CSF from the same patients was performed.

Among the most interesting findings of the CSF analysis was that some differentially charged protein isoforms of ApoA-1, ApoE, ApoJ, and ApoH are differentially expressed. This observation prompted the question: is it possible that diseases and biologic processes leave a trace of their causes and effects in the charge-isoform distribution pattern? To explore this observation further, we performed analysis of the charged isoforms of ApoJ, ApoH, ApoA-1, and ApoE in the blood of AD patients, compared with age- and sex-matched controls. Analysis of the ApoE charge-isoform distributions was sufficient to indicate that a distribution is unique to the diseased population compared with the control population, and that the charge-isoform distribution also carries sufficient information to determine the *APOE* genotype of the corresponding patients.

To test the validity of our results, we requested completely de-identified samples from the Knight Alzheimer’s Disease Research Center (Washington University, St. Louis, MO, USA). By using the distribution pattern of the charge-isoforms of ApoE, we determined the correct AD status in 16 of the 18 samples. We also were able to determine correctly the *APOE* genotype of the patients.

Additional analysis of ApoE charge-isoforms was sought by using quantitative proteomics based on spectral counting [39,40]. This procedure confirmed our previous observations [19] that the total amount of ApoE does not display changes, contrary to variations in expression of individual charge-isoforms. Despite extensive analysis, our MS procedures failed to determine the nature of the ApoE modifications that may be responsible for these variations in isoelectric focusing; however, not knowing the nature of the modifications does not diminish our claim that the charge-isoform distribution could be a biomarker for AD. As extensively demonstrated in this report, what distinguishes AD from non-AD samples is the distribution of the isoforms in an isoelectric analysis, not the nature of the modifications.

The results presented here have notable advantages over current biomarkers used by others in the scientific community: (a) these observations are made on human blood; (b) we target only one molecule for analysis instead of tens of compounds, which should make the diagnostic assays faster and less expensive; (c) these results can be implemented on high-throughput platforms either by optimizing our approach, implementing MS-based characterization of ApoE charge-isoforms, by using antibodies specific for the different ApoE charge-isoforms, or by developing fluorescent dyes specific for each of the unique charge-isoforms, (d) the methods presented here can be carried out in less than 4 hours and cost less than $200, making this approach a viable method for the diagnosis of this and other diseases, and (e) the use of bioinformatics to develop pattern-recognition software for analysis of charge-isoform distributions associated with diseases and different biologic states should provide a great opportunity for diagnosis and prognosis, and for determining disease initiation and progression.

## Conclusions

We report here the use of ApoE charge-isoforms as a method to determine the presence of AD in blood extracts. Our method is simple, requires little sample (less than 40 μl of blood plasma), and can be carried out in less than a day, without requiring the presence of the patient or expensive and sophisticated equipment. As indicated in our data, diagnosis from *APOE 3/3* patients can be made with high accuracy. Further studies should include significant numbers of *APOE 4/4* and *APOE 2/2* samples to create a robust reference panel applicable to any *APOE* genotype. Studies are also needed to validate these markers for the determination of the onset and the progression of the disease, as well as the effectiveness of therapeutic treatments.

## Abbreviations

2D DIGE: Two dimensional difference gel electrophoresis; 2D mxWb: two-dimensional multiplexed Western blotting; AD: Alzheimer’s disease; ApoE: apolipoprotein E; BADRC: Bryan Alzheimer’s Disease Research Center; BCA: bicinchoninic acid; CID-MS/MS: collision-induced dissociation tandem mass spectrometry; CSF: cerebrospinal fluid; DSM-IVR: Diagnostic and Statistical Manual of Mental Disorders, 4th edition; EDA: extended data analysis; EOFAD: early-onset familial Alzheimer’s disease; FDR: false discovery rate; IEF: isoelectric focusing; INDEC: Instituto Neurologico de Colombia (Colombian Neurological Institute); KADRC: Knight Alzheimer’s Disease Research Center; LC-MS/MS: liquid chromatography – tandem mass spectrometry; LOAD: late-onset Alzheimer’s disease; MALDI TOF/TOF: matrix-assisted laser desorption ionization time-of-flight mass spectrometry; MARS: Multiple Affinity Removal System; MDLC: multidimensional liquid chromatography; MIPS: monoisotopic precursor ion selection; MS: mass spectrometry; MS/MS: tandem mass spectrometry; PCA: principal component analysis; PIR: Protein information resource; PTM: posttranslational modification.

## Competing interests

The authors have no competing interests.

## Authors’ contributions

OA: Designed and supervised the studies, analyzed the data, and wrote the manuscript. CO: Collected data, helped analyze the results, and reviewed the manuscript; RD: Collected data, helped analyze the results, and reviewed the manuscript. AC: Collected data and reviewed the manuscript. HG: Performed MS-based label-free quantitative proteomics and reviewed the manuscript. The authors read and approved the final manuscript.

## Supplementary Material

Additional file 1 Differential protein expression in CSF of AD samples compared with non-AD matched controls. Click here for file
