# Supplementary material for: Differentially charged isoforms of apolipoprotein E from human blood are potential biomarkers of Alzheimer’s disease
Source: Alzheimers Res Ther. 2014 Jul 14;6(4):43. doi: 10.1186/alzrt273 (PMC4255367; doi:10.1186/alzrt273)
Supplement: Additional file 1 — Differential protein expression in CSF of AD samples compared with non-AD matched controls. [file alzrt273-S1.pdf]

A	B	C	D	E	F	G	H	I	J	K	L	M
	33N vs. 33D		33N vs. 44D		33D vs. 44D							
Spot No.	ANOVA	Avg Ratio	ANOVA	Avg Ratio	ANOVA	Avg Ratio	Protein Name (from Uniprot)	Accession Number	Molecular weight	Peptide count	MS & MS/MS score	Peptide sequence (ion score)
551	0.04	-1.46										
964	0.0054	1.45										
1133	0.015	1.66										
1138	0.032	1.71										
1202	0.015	-1.37										
1251	0.0093	1.44										
1270	0.015	-1.32										
1320	0.018	-1.29										
1326	0.014	-1.6										
1368	0.012	-1.45										
1447	0.0059	-1.67										
1449	0.015	-1.64										
1530	0.031	1.87										
1949	0.022	1.64										
1956	0.036	2.93										
430			0.0014	-1.56								
439			0.015	1.47								
477			0.0048	-1.29								
548			0.02	1.39								
881			0.035	1.41								
1005			0.013	1.74								
1168			0.046	1.15								
1247			0.016	-1.34								
1299			0.023	-1.5								
1308			0.024	-1.5								
1331			0.037	-1.54								
1341			0.045	-1.49								
1358			0.031	-4.53								
1431			0.0054	-1.66								
1484			0.015	-1.44								
1501			0.023	1.77								
1532			0.0051	2.07								
1552			0.019	1.85								
1557			0.02	1.57								
1559			0.02	1.44								
1571			0.0052	-1.26								
1611			0.045	-1.32								
1827			0.0017	2.35								
1846			0.032	1.56								
1864			0.014	1.75								
1878			0.0044	1.7								
1881			0.00093	2.05								
1896			0.01	1.88								
1897			0.0084	1.74								
1901			0.0038	1.79								
1904			0.0053	1.83								
1909			0.017	-2.88								
1924			0.00058	1.72								
1926			0.028	1.52								
1932			0.014	1.54								
1937			0.0098	1.54								
2027			0.012	-1.36								
151					0.031	1.32						
349					0.014	-1.18						
698					0.026	-1.23						
866					0.0087	1.62						
898					0.0012	1.42						
911					0.027	1.77						
955					0.0076	1.34						
976					0.026	1.3						
1013					0.018	1.54						
1028					0.015	1.3						
1097					0.0071	1.24						
1167					0.0073	-1.34						
1184					0.03	1.6						
1360					0.0035	-1.18						
1580					0.021	1.27						
1590					0.027	1.3						
1634					0.01	1.64						
1666					0.021	1.74						
1689					0.02	1.92						
1746					0.029	1.51						
2053					0.0021	-1.84						
a	b	c	d	e	f	g						
204	0.012	1.57	0.0007	1.74								
429	0.0049	-1.29	0.0014	-1.37			Serum Albumin, chain A	**P02768**	65694.5	19	289	187
513	0.0072	-1.55	0.00056	-1.61								
526	0.00088	-1.66	0.0093	-1.47								
541	0.0073	-1.53	0.012	-1.5			Transferrin variant	**Q53H26**	77029.6	15	270	203
561	0.0077	2.75	0.0071	2.4								
575	0.0072	-1.22	0.0062	-1.18			alpha 1-B glycoprotein	**P04217**	51908.4	12	507	441
579	0.015	-1.31	0.0054	-1.31			alpha 1-B glycoprotein	**P04217**	51908.4	11	465	408
614	0.036	1.7	0.0037	1.64			Transferrin variant	**Q53H26**	77029.6	27	701	509
848	0.02	1.29	0.014	1.6								
990	0.00066	1.67	0.041	1.6			Apolipoprotein H (beta-2-glycoprotein 1)	**P02749**	38272.7	11	175	114
1000	0.042	1.29	0.0073	1.73			Tubulin beta - 2A chain	**Q13885**	49884	14	179	83
1008	0.00063	2.94	0.018	2.62			Keratin type II, cytoskeletal 1	**P04264**	65846.9	19	286	166
1017	0.016	1.86	0.0087	2.26			Alpha-1-antitrypsin (Serpin A1)	**P01009**	46707	23	750	532
1096	0.0042	1.51	0.018	1.47								
1110	0.021	1.18	0.033	1.19								
1118	0.0025	1.54	0.0002	1.99			Pigment epithelium-derived factor (Serpin F1)	**P02768**	46372.4	11	205	140
1125	0.00074	2.45	0.0017	2.26			Pigment epithelium-derived factor (Serpin F1)	**P02768**	46372.4	15	436	326
1126	0.0025	2.03	0.0027	1.91			Pigment epithelium-derived factor (Serpin F1)	**P02768**	46372.4	14	222	99
1141	0.0002	2.07	0.038	1.97			Pigment epithelium-derived factor (Serpin F1)	**P02768**	46372.4	14	409	298
1148	0.0004	2.36	0.022	2.59								
1172	0.0084	1.52	0.00097	1.53								
1188	0.032	1.26	0.004	1.23			Serum Albumin, Chain A	**P02768**	47329.7	11	210	112
1268	0.0062	-1.38	0.0069	-1.55			Glutamine synthetase	**P15104**	42037.3	15	307	147
1278	0.0046	-1.46	0.037	-1.4			Creatine kinase B-type	**P12277**	42591.3	18	414	266
1313	0.000041	-1.88	0.0081	-1.51								
1324	0.01	-1.49	0.023	-1.53			Fructose-bisphosphate aldolase A	**P04075**	39307.3	9	158	78
1332	0.042	-1.28	0.011	-1.49			Aspartate aminotransferase, cytoplasmic	**P17174**	46106.5	14	233	96
1359	0.031	-6.93	0.038	-6.5								
1363	0.0046	-1.27	0.00067	-1.41			Aspartate aminotransferase, cytoplasmic	**P17174**	46106.5	9	99	45
1397	0.021	-1.48	0.0022	-1.55			Aging-associated gene 9 protein	**Q2TSD0**	36026.4	5	213	183
1451	0.0015	-2.05	0.0023	-1.75			Glyceraldehyde-3-phosphate dehydrogenase	**P04406**	36030.4	4	150	113
1454	0.029	-1.45	0.028	-1.42								
1456	1.9E-06	-1.92	0.00025	-1.67			Glyceraldehyde-3-phosphate dehydrogenase	**P04406**	36030.4	12	371	291
1469	0.0031	-1.58	0.0058	-1.52			Glyceraldehyde-3-phosphate dehydrogenase	**P04406**	36030.4	10	318	223
1470	0.0061	-1.8	0.0026	-1.72								
1472	0.003	2.01	0.00027	2.01			Clusterin, ApoJ	**P10909**	48772.1	15	140	61
1493	0.012	1.98	0.0048	1.85								
1500	0.006	2.45	0.00081	2.24								
1516	0.0065	1.52	0.0015	1.9								
1521	0.012	2.14	0.00043	3.34			Apolipoprotein E4	**P02649**	36184.9	11	95	46
1523	0.00084	1.78	2.3E-06	2.17			Clusterin, ApoJ	**P10909**	16266.8	4	97	52
1527	0.0022	2.02	0.00008	2.42			Apolipoprotein E4	**P02649**	36184.9	19	485	356
1535	0.0044	1.85	0.00012	1.94			Complement component 4A	**Q5JNX2**	29598.7	9	216	170
1545	0.038	1.61	0.039	1.72								
1554	0.015	2.05	0.0023	2.58			Transthyretin	**P02766**	12470.3	6	189	119
1581	0.0058	1.81	0.021	1.78								
1779	0.029	-1.98	0.015	-2.14			Ig Kappa chain C region; IGKC	**P01834**	65694.5	17	108	36
1845	0.045	1.45	0.0051	1.51			Prostaglandin-H2- D-isomerase; cerebrin 28	**P41222**	21015.3	5	130	98
1860	0.027	1.8	0.0025	1.92								
1900	0.022	1.36	0.00005	1.48								
1912	0.025	-1.78	0.016	-1.84			Apolipoprotein A-1	**P02647**	28061.5	23	380	144
1921	0.0074	1.62	0.00057	1.82								
1980	0.012	2.14	0.019	2.1								
1982	0.011	1.66	0.0022	1.62			Superoxide dismutase (Mn), mitochondrial	**P04179**	24706.6	4	98	49
1987	0.0083	1.79	0.005	1.72								
2067	0.00043	-1.95	8.9E-06	-2.09			Apolipoprotein A-1	**P02647**	28061.5	21	721	504
2068	0.0031	-2.25	0.00071	-2.24			Apolipoprotein A-1	**P02647**	28061.5	23	624	377
1315	0.0011	-1.64			0.033	1.39						
1573	0.011	1.53			0.018	-1.38						
592			0.0048	1.75	0.017	1.63						
596			0.00024	2.11	0.0038	1.69						
599			5.8E-06	2.14	0.000055	1.58						
1043			0.018	1.89	0.048	1.62						
1050			0.0063	2.06	0.014	1.83						
1114			0.0039	1.56	0.00086	1.24						
1403			0.021	-1.3	0.019	-1.15						
1549			9.5E-06	1.91	0.007	1.43						
1585			0.00054	1.65	0.0079	1.4						
1255	0.000067	-3.54	0.0057	-2.12	0.00056	1.67						
1546	0.00022	1.76	2.4E-06	4.26	0.00032	2.43						

H: Protein named as sugegsted by Uniprot; http://www.uniprot.org/
I: Protein accession # from Uniprot; http://www.uniprot.org/
J: Molecular weight from Uniprot
K: Peptide count: Number of peptides that matched the theoretical digest of the primary protein identified.
L: MS & MS/MS score: score of the quality of the peptide mass fingerprint and the quality of the MS/MS peptide fragmnet. L>=95 is considered significant.
M: Peptide sequence ion score: score of the quality of the MS/MS peptide fragment. Scores of 20 or greater are considered significant.
